# Movement Patterns and Muscular Function Before and After Onset of Sports-Related Groin Pain: A Systematic Review with Meta-analysis

**DOI:** 10.1007/s40279-016-0523-z

**Published:** 2016-05-03

**Authors:** Paulina Kloskowska, Dylan Morrissey, Claire Small, Peter Malliaras, Christian Barton

**Affiliations:** 1 Sport and Exercise Medicine Research Centre, School of Allied Health, La Trobe University, Melbourne, Australia; 2 Sports and Exercise Medicine, William Harvey Research Institute, Bart’s and the London School of Medicine and Dentistry, Queen Mary University of London, Mile End Hospital, Bancroft Road, London, E1 4DG UK; 3 Pure Sports Medicine, London, UK; 4 Complete Sports Care, Melbourne, VIC Australia; 5 Physiotherapy Department, Barts Health NHS Trust, London, UK

## Abstract

**Background:**

Sports-related groin pain (SRGP) is a common entity in rotational sports such as football, rugby and hockey, accounting for 12–18 % of injuries each year, with high recurrence rates and often prolonged time away from sport.

**Objective:**

This systematic review synthesises movement and muscle function findings to better understand deficits and guide rehabilitation.

**Study Selection:**

Prospective and retrospective cross-sectional studies investigating muscle strength, flexibility, cross-sectional area, electromyographic activation onset and magnitude in patients with SRGP were included.

**Search Methods:**

Four databases (MEDLINE, Web of Knowledge, EBSCOhost and EMBASE) were searched in June 2014. Studies were critiqued using a modified version of the Downs and Black Quality Index, and a meta-analysis was performed.

**Results:**

Seventeen studies (14 high quality, 3 low quality; 8 prospective and 9 retrospective) were identified. *Prospective findings*: moderate evidence indicated decreased hip abduction flexibility as a risk factor for SRGP. Limited or very limited evidence suggested that decreased hip adduction strength during isokinetic testing at ~119°/s was a risk factor for SRGP, but no associations were found at ~30°/s or ~210°/s, or with peak torque angle. Decreased hip abductor strength in angular velocity in ~30°/s but not in ~119°/s and ~210°/s was found as a risk factor for SRGP. No relationships were found with hip internal or external rotation range of movement, nor isokinetic knee extension strength. Decreased isokinetic knee flexion strength also was a potential risk factor for SRGP, at a speed ~60°/s. *Retrospective findings*: there was strong evidence of decreased hip adductor muscle strength during a squeeze test at 45°, and decreased total hip external rotation range of movement (sum of both legs) being associated with SRGP. There was strong evidence of no relationship to abductor muscle strength nor unilateral hip internal and external rotation range of movement. Moderate evidence suggested that increased abduction flexibility and no change in total hip internal rotation range of movement (sum of both legs) were retrospectively associated with SRGP. Limited or very limited evidence (significant findings only) indicated decreased hip adductor muscle strength during 0° and 30° squeeze tests and during an eccentric hip adduction test, but a decrease in the isometric adductors-to-abductors strength ratio at speed 120°/s; decreased abductors-to-adductors activation ratio in the early phase in the moving leg as well as in all three phases in the weight-bearing leg during standing hip flexion; and increased hip flexors strength during isokinetic and decrease in transversus abdominis muscle resting thickness associated with SRGP.

**Conclusions:**

There were a number of significant movement and muscle function associations observed in athletes both prior to and following the onset of SRGP. The strength of findings was hampered by the lack of consistent terminology and diagnostic criteria, with there being clear guides for future research. Nonetheless, these findings should be considered in rehabilitation and prevention planning.

## Key Points

**Table:** 

There are a number of movement and muscular function differences between healthy athletes and those suffering from sports-related groin pain (SRGP), which exist prior, and subsequent, to symptom onset.
In screening programmes the main focus should be to address hip adductor weakness, and consideration should also be given to addressing any hip abductor and knee flexor strength.
In planning rehabilitation, adductor strengthening as well as increasing hip internal and external range of movement should be the main focus; additionally, the balance between hip adductors and abductors activation and strength should be carefully assessed and managed.

## Introduction

Sports-related groin pain (SRGP) is a common clinical entity, accounting for 12–16 % of all sports injuries [1, 2]. It is particularly prevalent in sports involving rotation and cutting movements, such as football, rugby and hockey [3]. It is often associated with prolonged time away from sport [4, 5] and therefore considered a significant problem in professional sport.

The difficulties in diagnosis and treatment of SRGP are partly caused by the lack of consensus amongst researchers and clinicians in the classification of the functional anatomy of the area and the large range of diagnostic terms used [6]. Patients experiencing SRGP are often ‘diagnosed’ with osteitis pubis, adductor tendinopathy, sports hernia, Gilmore’s groin as well as iliopsoas-, rectus abdominis- and adductor-related muscular disorders. Various underlying tissue pathologies are likely to coexist [7] and there is a lack of clinical or imaging tests with high levels of sensitivity or specificity. A recently published Doha agreement [8] classified groin symptoms into four main sub-groups proposing a clear division between the hip-related pathologies from other (‘defined’) pathologies such as adductor-, iliopsoas-, inguinal- and pubic-related pain. It may be useful to implement this classification in further research on SRGP by dividing the study participants according to the sub-diagnoses defined by the Doha agreement. This would enable future work to determine whether these different diagnoses may influence the biomechanical signatures of SRGP, and may potentially reduce the variability associated with different sources of groin symptoms. However, a majority of studies investigating the biomechanical factors associated with SRGP were published prior to the Doha agreement meeting. Despite a lack of compliance with the proposed classification and exact diagnosis of these study participants, the results of different studies are generally consistent. These biomechancial similarities, despite varied diagnostic criteria, suggest that diagnostic precision may not be critical when considering the biomechanical determinants of SRGP.

Our review will therefore include all sub-diagnoses of groin pain, gathered under the umbrella term of SRGP. Further, we will consider movement and muscle function factors for specific tissue diagnoses where these are clear, but also across the SRGP group to identify common biomechanical patterns.

Two systematic reviews [9, 10] that have been published on the effectiveness of conservative therapy in SRGP have identified a paucity of high-quality research in this area. Both reviews indicate that regardless of the underlying initial pathology of the groin pain, active rehabilitation including flexibility, stretching and strengthening exercises of the pelvic girdle and hip muscles is critical in effective management. Studies supporting active rehabilitation for SRGP tend to focus on hip adductor and abdominal muscle strengthening [4, 5]. However, the sports specificity of these elements is limited. Although some proposed rehabilitation strategies have good long-term outcomes [11], the recurrence rate of groin symptoms is still relatively high [1, 2] suggesting that current rehabilitation strategies may not fully address deficits in the neuromuscular system. This systematic review and meta-analysis will provide evidence related to movement and muscle function deficits in athletes with SRGP, with the aim of providing a useful guide for clinicians and researchers developing and evaluating rehabilitation and prevention programmes.

## Methodology

### Inclusion and Exclusion Criteria

Prospective and retrospective cross-sectional (i.e. case–control) studies investigating movement and muscle function variables associated with chronic groin pain published in English from database inception to November 2015 were included. Groin pain diagnostic labels included ‘adduction-related groin pain’, ‘osteitis pubis’, ‘pubialgia’, ‘pubalgia’, ‘sports hernia’ or ‘adductor tendinopathy’. Only participants whose groin pain was associated with playing sports were included. Biomechanical terms included strength, flexibility (range of motion), muscle activation magnitude and timing, muscle size and cross-sectional area. Measurement techniques included magnetic resonance imaging, ultrasound, electromyography, dynamometer or physical examination.

Single-case studies, cadaver studies, studies on healthy participants only and studies without a control group were excluded. Studies including participants diagnosed with true hernias, and hip joint, thoracic or lumbar spine pathology were excluded from the review.

### Search Strategy and Review Process

A reproducible search strategy was created by three reviewers (PK, CB and DM). The search terms combined muscle features or measurement tools (“strength” OR “flexibility” OR “cross-section*” OR “onset” OR “activation” OR “range of motion” OR “ROM” OR “EMG” OR “electromyograph*” OR “ultrasound*” OR “dynamometer” OR “MRI” OR “magnetic resonance imaging” OR “ultrasonograph*” OR “US”) and diagnostic terms (“groin pain” OR “chronic groin pain” OR “osteitis pubis” OR “pubialgia” OR “pubalgia” OR “adductor pain” or “adductor tendin*” OR “adductor tendon*” OR “adductor* strain” OR ““adductor*” injur*”). MEDLINE, Web of Knowledge, EMBASE and EBSCOhost databases were searched, using keywords wherever possible.

Retrieved studies were entered into Endnote (Thomson, Reuters, Carlsbad, California, USA) and duplicates deleted. Titles and abstracts were screened against the inclusion and exclusion criteria by two independent reviewers (PK and CS). Where necessary, abstracts and full texts were obtained to make a final decision. A third reviewer (CB) was available to reach consensus if there were any conflicts. The reference lists of included studies were searched and citation tracking performed via Google Scholar for additional relevant studies.

### Quality Assessment and Study Analysis

A modified version of the Downs and Black Quality Index was applied by two independent reviewers (PK and CS) to assess the quality of included studies. A third reviewer was available to resolve differences (DM). Irrelevant questions referring to intervention trials were excluded from the questionnaire. Fifteen relevant questions built up a modified version of the Downs and Black Quality Index, with a maximum score of 16 points [12]. Papers were considered as high-quality studies (HQS) when scored above 10 (inclusive) points and low-quality studies (LQS) when scored below 10 points, following Barton et al. [12].

### Data Extraction and Analysis

Characteristics of the study participants (number, type and level of sport, age, height and weight), diagnosis of the symptomatic patients, task (if relevant), muscle and/or muscle group, diagnostic tool, and results of symptomatic and control group were extracted from the selected articles (Table 1). Means and standard deviations were extracted to enable calculation of standard mean differences (SMDs). Where the presentation of the data was not adequate to calculate SMDs, corresponding authors were contacted by email in an attempt to obtain the data. In one case [13] where the standard deviation was not published, it was calculated by the authors of this review as the paper included individual participant values for variables measured. Where possible, data were pooled for common measurement features of given muscle groups to establish the levels of evidence. If results were not presented nor obtained from authors in a format allowing data pooling, it was omitted in the meta-analysis. If only one study investigated given muscle characteristics, SMD was calculated from the result presented in this study. This analysis is more stringent than statistics commonly used in individual studies (such as the *t* test). It might, therefore, show different results to those reported previously within a specific study.

**Table 1:** Participants characteristics

References	Type of study	Diagnosis	*N* (SRGP:C)	Type of sport	Level of sport	Age, years [mean (SD), range or SMD (95 % CI)]	Weight, kg [mean (SD) or SMD (95 % CI)]	Height, cm or m [mean (SD) or SMD (95 % CI)]
Arnason et al. [17]	Prospective cohort	Groin strain	17:281	Icelandic football (soccer)	Elite league and first division	SRGP: 25.1 (1.2); C: 24.0 (0.2)	SRGP: 79.1 (1.2); C: 76.4 (0.4)	SRGP: 183.0 (1.4); C: 180.5 (0.4)
Cowan et al. [19]	Retrospective case–control	Long-standing groin pain	10:12	Australian Rules Football	Elite or sub-elite	SRGP: 26 (7); C: 25 (6)	SRGP: 78.1 (8.4); C: 76.8 (11.3)	SRGP: 180.7 (7); C: 176.5 (7.9)
Crow et al. [20]	Prospective	Groin injury	12:12	Australian Rules Football	Elite	16–18	N/R	N/R
Emery and Meeuwisse [33]	Prospective cohort	Groin strain injury	204:1088	Canadian National Hockey League	Professional	N/R	N/R	N/R
Engebretsen et al. [29]	Prospective cohort	Groin injury	51:457	Football (soccer)	Amateur	N/R	N/R	N/R
Ibrahim et al. [13]	Prospective	Adductor strain	8:79	Australian Rules Football	Professional	N/R	N/R	N/R
Jansen et al. [21]	Retrospective case–control	Adduction-related groin pain	42:23	Various (football, soccer, running, field hockey, cycling, korfball, fitness, rugby, swimming, speed skating)	Amateur	R SRGP: 24.8 (6.9); L SRGP: 28.2 (10.4); C: 23.9 (4.7)	R SRGP: 80.0 (9.2); L SRGP: 76.4 (11.8); C: 78.9 (6.8)	R SRGP: 184.4 (6.8); L SRGP: 181.4 (6.5); C: 183.7 (6.7)
Malliaras et al. [25]	Retrospective case–control	Groin pain	10:19	Australian Rules Football and soccer	Elite	SRGP: 17.3 (0.8); C: 17.1 (1.6)	SRGP: 78.5 (7.0); C: 77.1 (5.4)	SRGP: 184.4 (6.7); C: 183.9 (7.8)
Mens et al. [28]	Retrospective case–control	Adduction-related groin pain	44:44	Various (football, soccer, tennis, field hockey, basketball, fitness training, horseback riding, running)	Amateur	SRGP: 31.3 (28.1–34.6); C: 32.2 (30.0–35.4)	SRGP: 79.4 (76.3–82.5); C: 82.4 (79.5–85.3)	NR
Mohammad et al. [23]	Retrospective case–control	Osteitis pubis	20:20	Football (soccer)	N/R	SRGP: 19.94 (3.51); C: 20.78 (3.35)	SRGP: 70.91 (7.26); C: 71.33 (7.35)	SRGP: 176.16 (4.93); C: 176.00 (4.15)
Morrissey et al. [26]	Retrospective case–control	Chronic groin pain	09:09	Football code	Amateur	SRGP: 24 (3); C: 25 (2)	SRGP: 81 (4); C: 82 (3)	SRGP: 1.8 (0.1); C: 1.8 (0.1)
Nevin and Delahunt [27]	Retrospective case–control	Long-standing groin pain	18:18	Gaelic football	Club-level	SRGP: 23.89 (3.18); C: 23.83 (3.55)	SRGP: 80.28 (9.77); C: 72.28 (10.3)	SRGP: 1.79 (0.06); C: 1.80 (0.06)
O’Connor [30]	Prospective	Groin injury	21:72	Australian Rugby	Professional (first or reserve grade)	SRGP: 22.2 (2.9)^a^; C: 20.2 (4.5)^a^	SRGP: 90.5 (9.5)^a^; C: 84.7 (10.2)^a^	SRGP: 1.80 (0.13); C: 1.78 (0.06)
Thorborg et al. [18]	Cross-sectional	Adductor-related groin pain	21:16	Football (soccer)	Elite and sub-elite	SRGP: 24.5 (2.5); C: 22.9 (2.4)	SRGP: 74.6 (6.4); C: 78.6 (6.3)	SRGP: 179.8 (5.9); C: 179.8 (5.0)
Tyler et al. [22]	Prospective	Adductor strain	08:37	Ice hockey	Professional	N/R	N/R	N/R
Verral et al. [31]	Retrospective case–control	Chronic groin injury	47:42	Australian Rules Football and soccer	Professional	N/R	N/R	N/R
Verral et al. [32]	Prospective cohort	Chronic groin injury	04:25	Australian Rules Football	Professional	SRGP: 22.75 (1.70); C: 21.16 (0.63)	SRGP: 72.50 (3.28); C: 84.92 (1.99)	SRGP: 175.50 (2.33); C: 177.36 (6.82)

*SRGP* sports-related groin pain, *C* controls, *N/R* not reported, *SD* standard deviation, *R* right, *L* left, *SMD* standardised mean difference, *CI* confidence interval

^a^Significant difference between sports-related groin pain patients and control participants

If the results of a study were provided for both legs/both sides of the body, only data from the right or dominant side of the body were further calculated to maintain the data independence, as described or presented in previous studies [14, 15].

In studies reporting results from isokinetic measurements, originally reported radians per second (rad*s^−1^) were converted to degrees per second (°/s) to facilitate the delivery of the clinical implications.

Definitions for ‘levels of evidence’ were guided by recommendations made by van Tulder et al. [16]:

Strong evidence was defined as pooled results derived from three or more studies, including a minimum of two HQS, which are statistically homogenous (*p* > 0.05). Moderate evidence was defined as statistically significant pooled results derived from multiple studies, including at least one HQS, which are statistically heterogeneous (*p* < 0.05); or from multiple LQS which are statistically homogenous (*p* > 0.05). Limited evidence was defined as results from multiple LQS that are statistically heterogeneous (*p* < 0.05); or from one HQS. Very limited evidence was defined as results from one LQS. Conflicting evidence was defined as not significant pooled results, derived from multiple studies (regardless of quality), of which some may show statistical significance individually. These studies must be statistically heterogeneous (*p* < 0.05) that is, the results of studies are inconsistent.

## Results

**Fig. 1:**
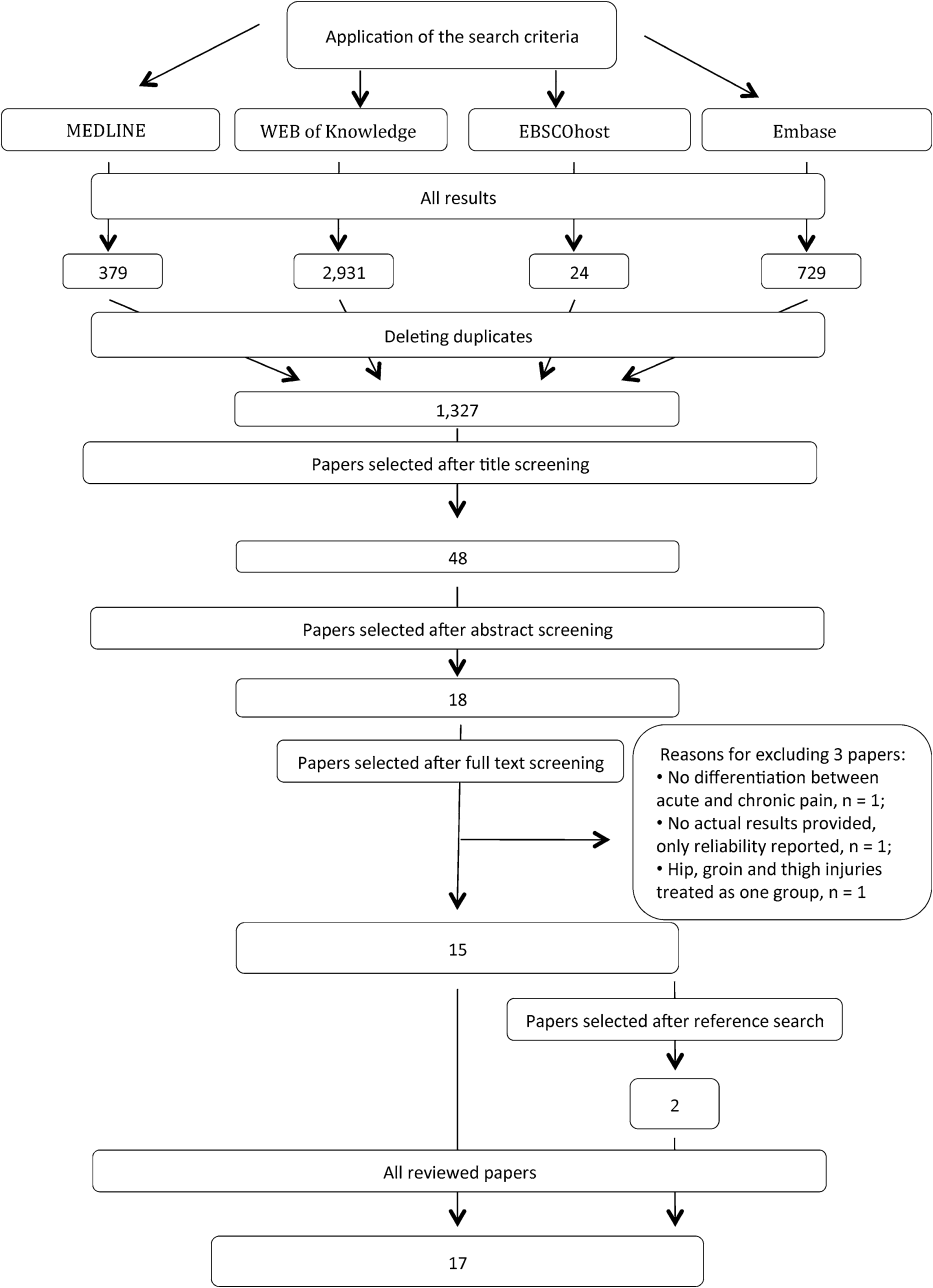
Flow chart showing studies inclusion and exclusion process for the review

Seventeen studies were included in the final yield. The search results from each database are shown in Fig. 1. Reference list screening of included studies identified two additional studies [17, 18] to the initial 15 included studies.

### Quality Assessment and Data Analysis

The details of the modified Downs and Black Quality Index results are shown in Table 2. The scores for the studies included in the review ranged between 8 and 15, with an average of 11. Of 17 included studies, 14 were HQS and 3 were LQS.

**Table 2:** Results of the quality assessment using a modified Downs and Black Quality Index [24]

D&B criterion References	(1)	(2)	(3)	(5)	(6)	(7)	(10)	(11)	(12)	(15)	(16)	(18)	(20)	(21)	(25)	Total	Study quality
Thorborg et al. [18]	1	1	1	2	1	1	1	1	0	1	1	1	1	1	1	15	HQS
Arnason et al. [17]	1	1	1	2	1	1	1	1	0	0	1	1	1	1	1	14	HQS
Cowan et al. [19]	1	1	1	2	1	1	1	0	0	0	1	1	1	1	1	13	HQS
Mens et al. [28]	1	1	1	1	1	1	1	0	0	1	1	1	1	1	1	13	HQS
Engebretsen et al. [29]	1	1	1	0	1	1	1	1	1	1	1	1	0	1	0	12	HQS
Malliaras et al. [25]	1	1	1	1	1	1	1	0	0	0	1	1	1	1	1	12	HQS
O’Connor [30]	0	1	1	2	1	1	1	0	0	0	1	1	1	1	1	12	HQS
Crow et al. [20]	1	1	1	0	1	1	1	0	0	0	1	1	1	1	0	10	HQS
Emery and Meeuwisse [33]	1	1	1	0	1	1	0	1	0	0	1	1	1	1	0	10	HQS
Ibrahim et al. [13]	1	1	1	0	1	0	1	1	1	0	1	0	1	1	0	10	HQS
Jansen et al. [21]	1	1	1	1	1	1	1	0	0	0	1	1	1	0	0	10	HQS
Morrissey et al. [26]	1	1	1	1	1	0	0	0	0	0	1	1	1	1	1	10	HQS
Tyler et al. [22]	1	1	1	1	1	0	1	0	0	0	1	1	1	1	0	10	HQS
Verral et al. [31]	1	1	1	0	1	1	1	0	0	0	1	1	1	1	0	10	HQS
Nevin and Delahunt [27]	1	1	1	1	0	1	0	0	0	0	1	1	1	0	1	9	LQS
Verral et al. [32]	1	1	0	1	1	1	1	0	0	0	1	1	0	1	0	9	LQS
Mohammad et al. [23]	1	1	0	1	0	1	1	0	0	0	1	1	0	0	1	8	LQS

(1) Clear aim/hypothesis, (2) clear outcome measures, (3) clear participant characteristics, (5) clear principal confounders, (6) clear study findings, (7) estimates of random variability provided, (10) probability values provided, (11) invited participants representative of entire population, (12) participants prepared to participate representative of entire population, (15) attempt to blind outcome measures, (16) no data dredging, (18) appropriate statistical tests, (20) valid and accurate outcome measures, (21) appropriate case–control matching, (25) adequate adjustment for confounding variables, *D&B* Downs and Black Quality Index, *HQS* high-quality study, *LQS* low-quality study

Where possible, the results of reviewed studies were pooled for analysis using Review Manager 5.2. Outcome values from a few papers were not reported and not obtainable despite contacting corresponding authors [19–23].

### Diagnostic Nomenclature

Reviewed studies used a variety of diagnostic terms including groin pain [25], chronic groin pain [26], long-standing groin pain [19, 27], adductor-related groin pain [18], adduction-related groin pain [21, 28], groin strain [17], groin injury [20, 29, 30], chronic groin injury [31, 32], adductor strain [13, 22], groin or abdominal strain injury [33] and osteitis pubis [23].

### Adductor Muscle Characteristics

#### Adductor Muscle Strength

Prospectively, four HQS [20, 22, 29, 30] reported a significant decrease of adductor muscle strength as a risk factor for SRGP, whilst one HQS reported adductor muscle strength was not associated with the risk of SRGP [33]. Four of the reviewed studies [22, 29, 30, 33] measured the difference in adductor muscle strength compared with the healthy controls, while one study [20] measured the decrease of adductor strength from a pre-season baseline measurement in athletes subsequently injured.

**Fig. 2:**
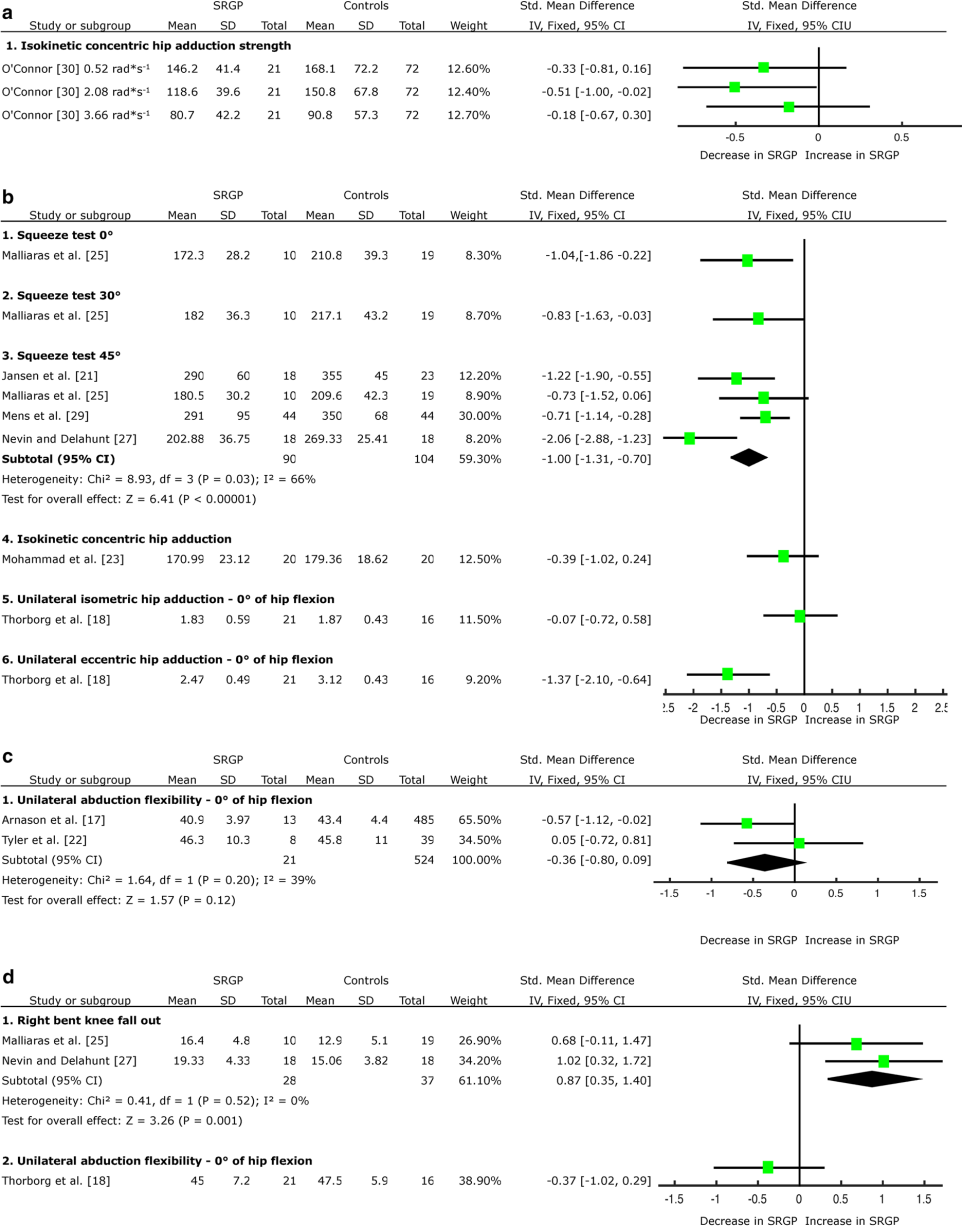

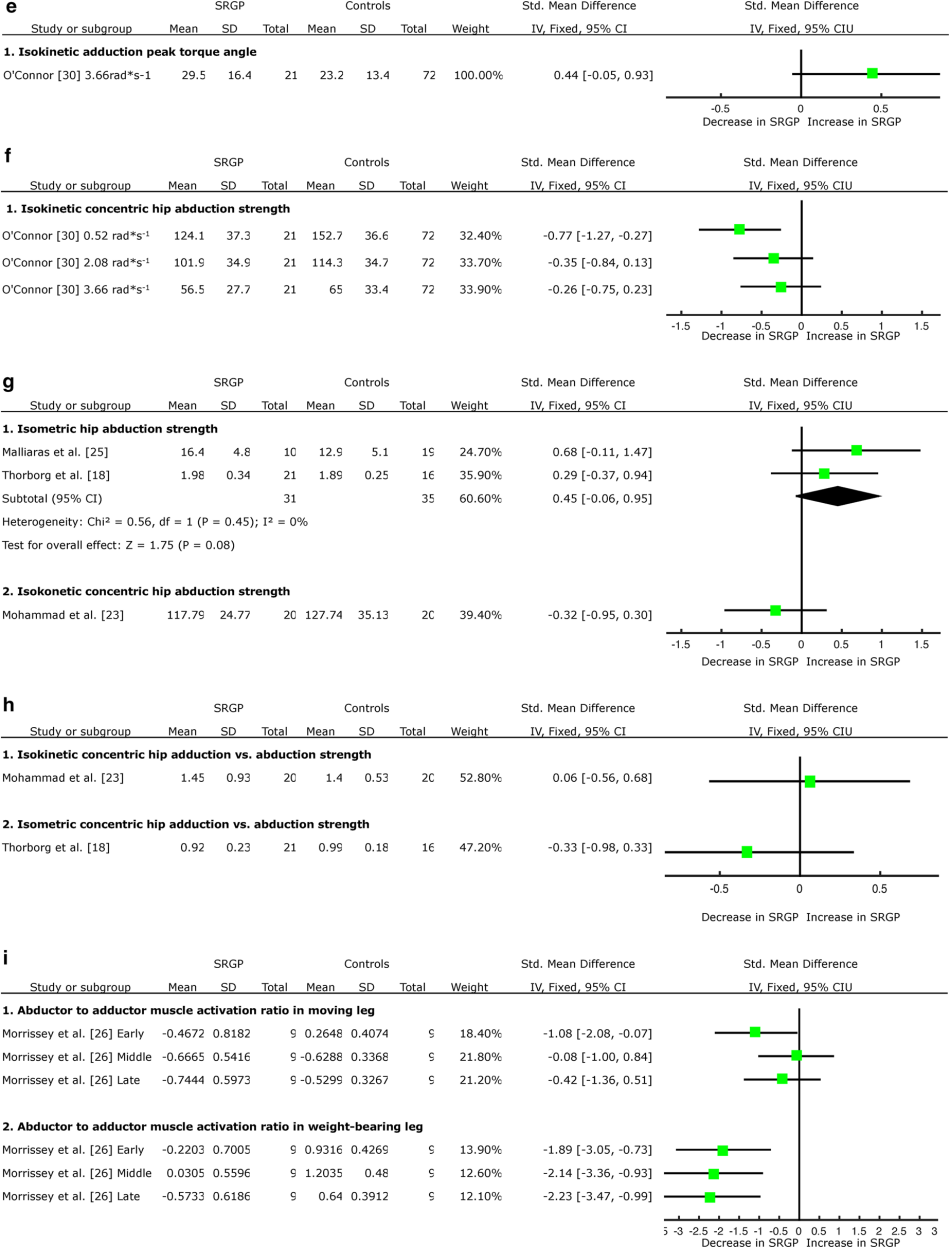
Forest plot detailing the analysis of movement and muscular functions in the coronal plane: **a** adductor muscle strength prospective results, **b** adductor muscle strength retrospective results, **c** abduction flexibility prospective results, **d** abduction flexibility retrospective results, **e** adduction peak torque angle retrospective results, **f** abductor muscle strength prospective results, **g** abductor muscle strength retrospective results, **h** adductor-to-abductor strength ratio retrospective results, and **i** abductor-to-adductor muscle activation ratio retrospective results. *SRGP* sports-related groin pain, *SD* standard deviation, *Std* standard, *IV* inverse variance, *CI* confidence interval

Three of the studies measured adductor strength preseason [22, 29, 33]. One study performed measurements weekly within season [20], and reported a significant decrease of adductor strength no sooner than 2 weeks pre-injury. Only one HQS [30] presented adequate data to complete the calculation of SMDs, which indicated limited evidence of decreased adductor muscle strength during the isokinetic test in angular velocity of 2.08 rad*s^−1^ (~119°/s) (SMD = −0.51, 95 % CI −1.00 to −0.02) as a risk factor for SRGP, but not in angular velocities of 0.52 rad*s^−1^ (~30°/s) (SMD = −0.33, 95 % CI −0.81 to 0.16) and 3.66 rad*s^−1^ (~210°/s) (SMD = −0.18, 95 % CI 0.67 to 0.30) (Fig. 2a). No indication was provided regarding when these measurements were taken.

Retrospectively, there was strong evidence emerging from three HQS [21, 25, 28] and one LQS [27] of the existing association between adductor muscle weakness during the squeeze test in 45° hip flexion and SRGP (SMD = −1.00, 95 % CI −1.31 to −0.70) (Fig. 2b). There was limited evidence from a single HQS of decreased adductor muscle strength during the squeeze test in 0° (SMD = −1.04, 95 % CI −1.86 to −0.22) and 30° (SMD = −0.83, 95 % CI −1.63 to −0.03) of hip flexion [25] (Fig. 2b); and during the eccentric adduction strength test [18] (SMD = −1.37, 95 % CI −2.10 to −0.64, Fig. 2b) associated with SRGP. Limited evidence emerged from one HQS of no difference in adductor muscle strength during the isometric adduction strength test [18] associated with SRGP; very limited evidence emerged from one LQS indicates adductor muscle strength during isokinetic measurements in angular velocity 2.1 rad*s^−1^ (~120°/s) is not a risk factor for SRGP development [23] (Fig. 2b).

#### Abduction Flexibility

Prospectively, three HQS [17, 22, 34] reported no change in abduction flexibility preceding the onset of SRGP. Two studies presented adequate data to complete the meta-analysis [17, 22], providing moderate evidence that abduction flexibility is not a risk factor for SRGP development (SMD = −0.36, 95 % CI −0.80 to 0.09, Fig. 2c).

Retrospectively, there was moderate evidence emerging from two HQS [18, 25] on an existing association between increased abduction flexibility during the bent knee fall-out test and SRGP (SMD = 0.87, 95 % CI 0.35 to 1.40, Fig. 2d). Limited evidence emerged from one HQS [18] of no change in abduction flexibility during the unilateral test in 0° of hip flexion and SRGP (Fig. 2d).

#### Adductor Muscle Peak Torque Angle

Prospectively, there was limited evidence from one HQS [30] that adductor muscle peak torque angle change in angular velocity of 3.66 rad*s^−1^ (~210°/s) is not a risk factor for SRGP development (Fig. 2e).

### Abductor Muscle Characteristics

#### Abductor Muscle Strength

Prospectively, there was limited evidence from one HQS [30] of a decrease in abductor muscle strength during the isokinetic test in angular velocity of 0.52 rad*s^−1^ (~30°/s) (SMD = −0.77, 95 % CI −1.27 to −0.27) as a risk factor for SRGP development, but not in angular velocities of 2.08 rad*s^−1^ (~119°/s) and 3.66 rad*s^−1^ (~210°/s) (Fig. 2f).

Retrospectively, there was strong evidence emerging from two HQS [18, 25] of no change in abductor muscle strength during isometric unilateral measurements; and very limited evidence emerging from one LQS [23] of no difference in abductor muscle strength during isokinetic measurements in angular velocity 2.1 rad*s^−1^ (~120°/s), associated with SRGP (Fig. 2g).

### Relation Between Adductor and Abductor Muscles

#### Muscle Strength Ratios

Prospectively, one HQS [22] reported a decreased adductor-to-abductor muscle strength ratio as a risk factor for SRGP, but the format of data presentation was not adequate to complete the calculation of the SMD.

Retrospectively, there was limited evidence emerging from one HQS [18] and very limited evidence emerging from one LQS [23] of no change in isometric or isokinetic [in angular velocity 2.1 rad*s^−1^ (~120°/s)] adductor-to-abductor muscle strength ratio associated with SRGP (Fig. 2h).

#### Abductor-to-Adductor Muscle Activation Ratio

Retrospectively, one HQS [26] provided limited evidence of a decreased gluteus medius (GM)-to-adductor longus (AL) muscle activation ratio associated with SRGP in the moving leg during early (SMD = −1.08, 95 % CI −2.08 to −0.07), but not during middle or late phases of standing hip flexion movement (SHF) (Fig. 2i). The same study provided limited evidence of a decreased GM-to-AL muscle activation ratio associated with SRGP in the weight-bearing leg during early (SMD = −1.89, 95 % CI −3.05 to −0.73), middle (SMD = −2.14, 95 % CI −3.36 to −0.93) and late (SMD = −2.23, 95 % CI −3.47 to −0.99) phases of SHF (Fig. 2i).

### Hip Flexor Muscle Characteristics

#### Hip Flexor Muscle Strength

**Fig. 3:**
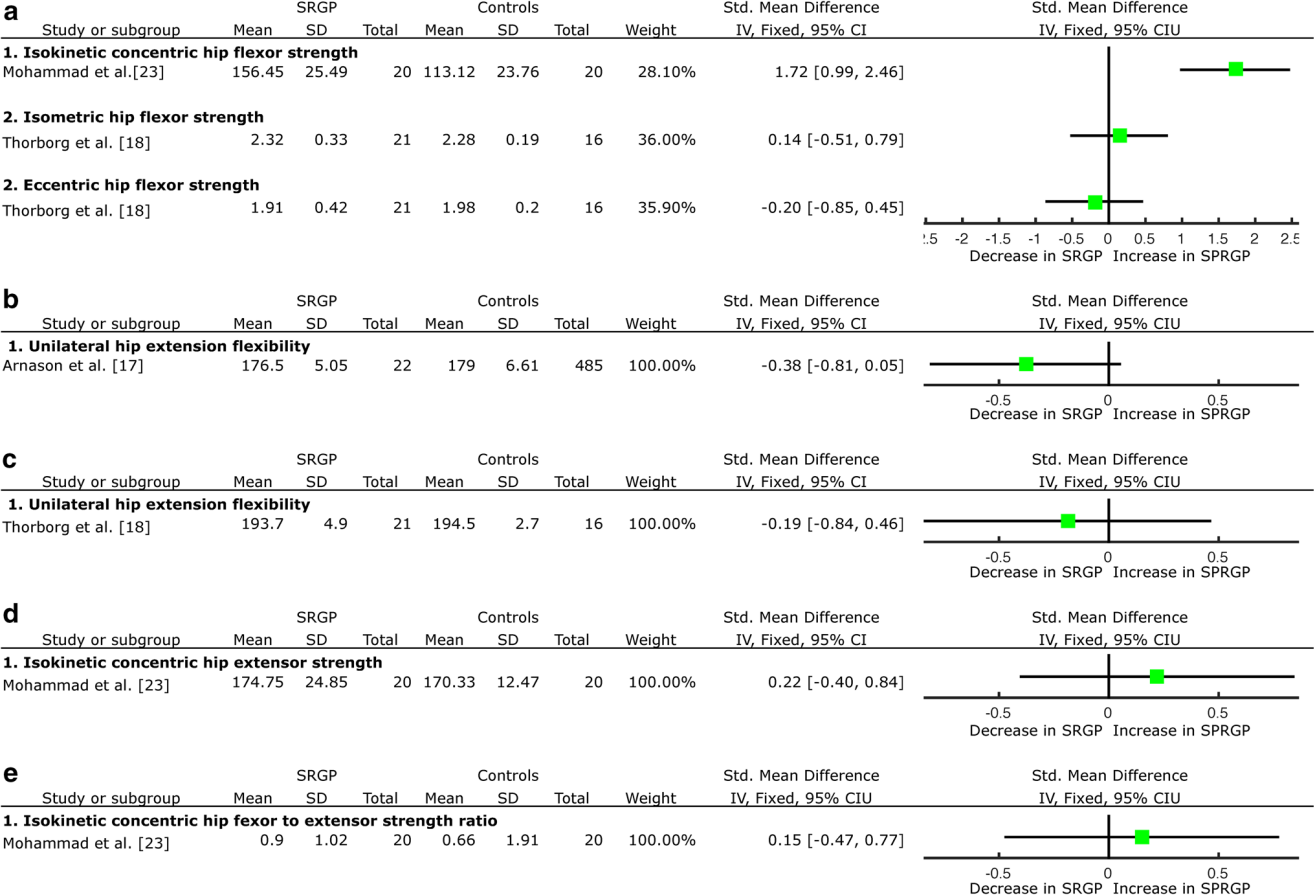
Forest plot detailing the analysis of movement and muscular functions in the sagittal plane: **a** flexor muscle strength retrospective results, **b** flexor muscle flexibility prospective results, **c** flexor muscle flexibility retrospective results, **d** extensor muscle strength retrospective results, and **e** flexor-to-extensor muscle strength ratio retrospective results. *SRGP* sports-related groin pain, *SD* standard deviation, *Std* standard, *IV* inverse variance, *CI* confidence interval

Retrospectively, there was very limited evidence provided by one LQS [23] of increased hip flexor muscle strength during the isokinetic test in angular velocity 2.1 rad*s^−1^ (~120°/s) associated with SRGP (SMD = 1.72, 95 % CI 0.99 to 2.46); and limited evidence emerging from one HQS [18] of no change in hip flexor strength during isometric and eccentric strength tests associated with SRGP (Fig. 3a).

#### Hip Extension Flexibility

Prospectively, there was limited evidence provided by one HQS [17] of no association between hip extension flexibility and the risk of SRGP development (Fig. 3b).

Retrospectively, there was limited evidence from one HQS [18] of no association between hip extension flexibility and SRGP (SMD = −0.19, 95 % CI −0.84 to 0.46, Fig. 3c).

### Hip Extensor Muscle Characteristics

#### Hip Extensor Muscle Strength

Retrospectively, there was very limited evidence emerging from one LQS [23] of no association between hip extensor muscle strength during the isokinetic test in angular velocity 2.1 rad*s^−1^ (~120°/s) and SRGP (SMD = 0.22, 95 % CI −0.40 to 0.84, Fig. 3d).

### Hip Flexor-to-Extensor Muscle Ratio

Retrospectively, there was very limited evidence emerging from one LQS [23] of no association between the hip flexor-to-hip extensor muscle strength ratio during isokinetic test in angular velocity 2.1 rad*s^−1^ (~120°/s) and SRGP (SMD = 0.15, 95 % CI −0.47 to 0.77, Fig. 3e).

### Hip Rotation Range of Movement

**Fig. 4:**
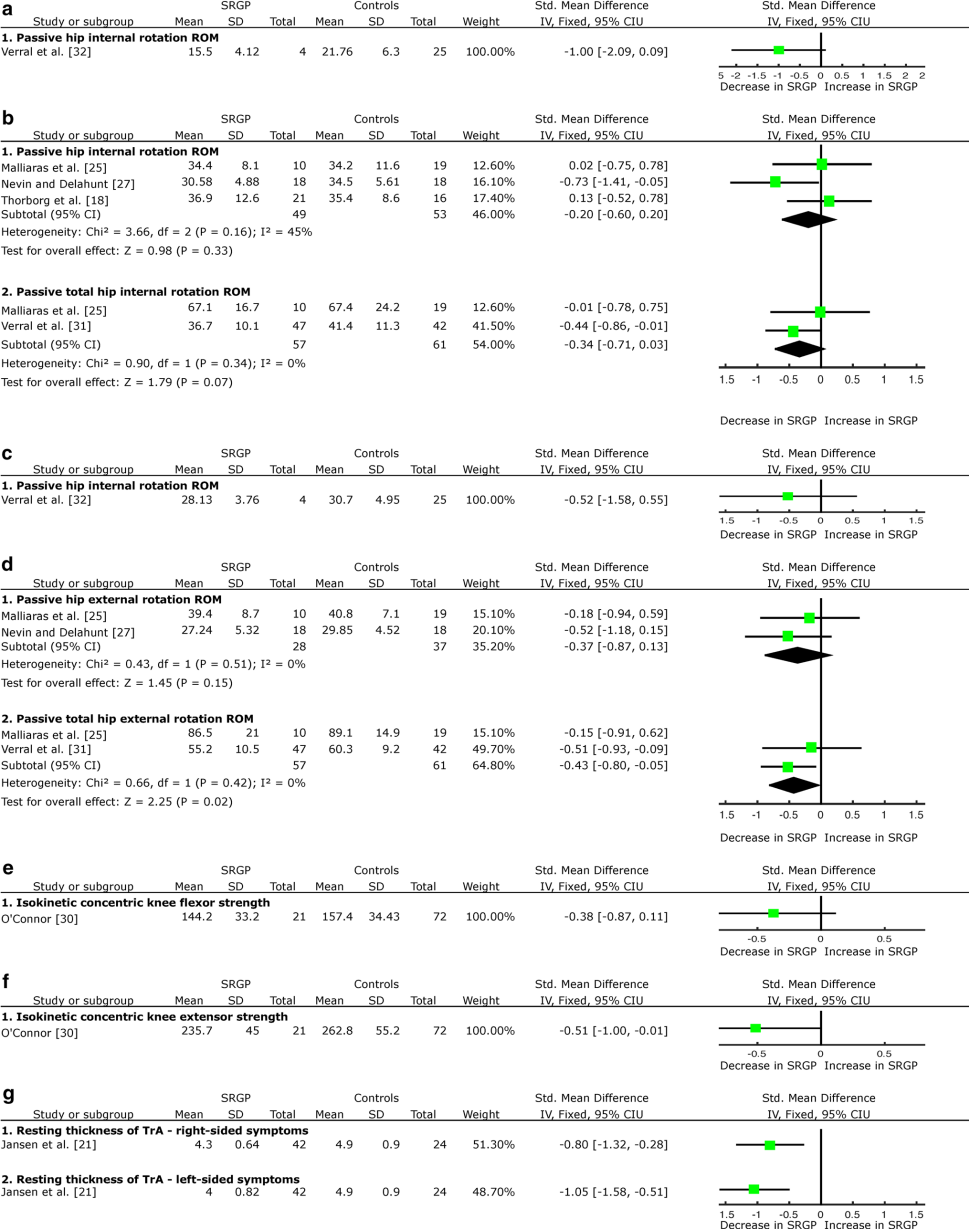
Forest plot detailing the analysis of other movement and muscular functions: **a** hip internal rotation ROM prospective results, **b** hip internal rotation ROM retrospective results, **c** hip external rotation ROM prospective results, **d** hip external rotation ROM retrospective results, **e** knee flexor muscle strength prospective results, **f** knee extensor muscle strength prospective results, and **g** transversus abdominis muscle thickness. *SRGP* sports-related groin pain, *SD* standard deviation, *Std* standard, *IV* inverse variance, *CI* confidence interval, *ROM* range of movement, *TrA* transversus abdominis muscle

Prospectively, there was very limited evidence from one LQS [32] that hip internal and external rotation range of movement (ROM) is not a risk factor for SRGP development (Fig. 4a, c).

Retrospectively, there was strong evidence emerging from two HQS [18, 25] and one LQS [27] on no difference in the unilateral hip internal rotation ROM; and strong evidence emerging from two HQS [25, 31] of no difference in the bilateral hip total internal rotation ROM (sum of both legs), associated with SRGP (Fig. 4b). There was moderate evidence emerging from one HQS [25] and one LQS [27] of no difference in the unilateral hip external rotation ROM; but strong evidence emerging from two HQS of decreased bilateral, total hip external rotation ROM (sum of both legs) associated with SRGP (SMD = −0.43, 95 % CI −0.80 to −0.05, Fig. 4d).

### Knee Muscle Characteristics

Prospectively, there was limited evidence from one HQS [30] that knee flexor muscle isokinetic strength measured in angular velocity measurements in angular velocity 1.04 rad*s^−1^ (~60°/s) is not a risk factor for SRGP (Fig. 4e). The same study provided limited evidence that the decreased, concentric, knee extensor muscle strength measured in angular velocity measurements of 1.04 rad*s^−1^ (~60°/s) is not a risk factor for SRGP (SMD = −0.51, 95 % CI −1.00 to −0.01, Fig. 4f).

### Abdominal Muscle Characteristics

Retrospectively, there was limited evidence from one HQS [21] of a decrease of transversus abdominis (TrA) muscle thickness at rest in participants with right-sided (SMD = −0.80, 95 % CI −1.32 to −0.28, Fig. 4g) and left-sided SRGP symptoms (SMD = −1.05, 95 % CI −1.58 to −0.51, Fig. 4g). One HQS [19] reported a delay in activation onset during the active straight leg raise task associated with SRGP, but adequate data were not available to complete SMD calculations.

One study [21] additionally reported no change in TrA thickness during the active straight leg raise (ASLR) and bilateral isometric adduction test; and internal and external oblique muscle thickness at rest, ASLR or bilateral isometric adduction associated with SRGP, but adequate data were not available to complete SMD calculations.

One study [19] reported no change in internal oblique and rectus femoris muscle activation onset timing during ASLR associated with SRGP, but adequate data were not available to complete SMD calculations.

## Discussion

This systematic review and meta-analysis synthesised 17 studies, including eight prospective and nine retrospective, which investigated changes in movement and muscle function in professional and amateur athletes with SRGP. Overall, there was conclusive evidence that measurable differences in movement and muscle function factors exist in athletes with SRGP, some of which may precede and increase the risk of developing injury. The findings should be considered by clinicians when designing rehabilitation and screening programmes.

There were some strong findings emerging from the evidence synthesis. The most notable, supported by strong or moderate evidence (Table 3), were retrospective associations between existing SRGP and adductor muscle weakness, increased abduction flexibility (bent knee fall out), and decreased internal and external rotation ROM. These results should be particularly considered when designing rehabilitation programmes for athletes with established SRGP. Prospectively, a paucity of evidence and data are available to complete the meta-analysis, but limited evidence indicates reduced hip adduction strength may be a risk factor for SRGP development. Additionally, it is worth noting that numerous studies also reported hip abductor strength deficits as a risk factor for SRGP development, but could not be included in the meta-analysis owing to a lack of reported data and response requesting additional data from corresponding authors. Nonetheless, hip abduction strength deficits should be particularly considered in screening programmes.

**Table 3:** Summary of the clinical implications emerging from this review

Clinical variable assessed		Finding	Implications for clinical practice	
Muscle group	Feature	Main result	Include in screening (prospective findings)	Include in rehabilitation (retrospective findings)
Adductor	Strength	Decrease in SRGP	✓	✓✓✓
	Flexibility	Increase in SRGP		✓✓
Abductor	Strength	Decrease in SRGP	✓	✓
Relationship between abductor and adductor muscles	Strength	Decrease in SRGP		✓
	Activation	Decrease in SRGP		✓
Hip flexor	Strength	Increase in SRGP		✓
Hip rotation ROM	Hip external rotation	Decrease in SRGP		✓✓✓
Knee flexor	Strength	Decrease in SRGP	✓	
Transversus abdominis	Thickness	Decrease in SRGP		✓

*SRGP* sports-related groin pain, *ROM* range of movement, ✓✓✓ indicates strong evidence, ✓✓ indicates moderate evidence, ✓ indicates limited or very limited evidence

### Methodological Considerations of Included Studies

There have been numerous attempts to introduce a common classification system for diagnosing SRGP [7, 28, 35], which we have not added to but have instead combined pragmatically to enable review. All but one study [23] provided clear diagnostic criteria. There was heterogeneity of SRGP definitions, with 11 subtly different diagnostic criteria being identified. This may have limited the strength of the review, but the similarities between classifications mean we were confident our review was sufficiently robust with each study using similar inclusion criteria regardless of the diagnostic term. For example, both Morrissey et al. [26] and Malliaras et al. [25] use an anatomical location of pain analysis alongside resisted movement tests and passive joint stress tests to differentially diagnose adductor tendinopathy with respect to hip joint pathology. They differ in that Malliaras et al. [25] additionally assessed the symptoms during a functional task such as agility drills, but these differences are relatively minor. Very similar inclusion criteria, based mainly on the palpatory pain of the adductor muscle, tendon or insertion area, and reproduction of symptoms during resisted hip adduction, were presented by Cowan et al. [19], Jansen et al. [21], Morrissey et al. [26] and Thorborg et al. [18]. Interestingly, the diagnostic term was different in all studies: long-standing groin pain [19], adduction-related groin pain [21], chronic groin pain [26] and adductor-related groin pain [18]. There is no question that initial recent attempts to establish an international consensus on groin pain nomenclature should reduce confusion and lack of agreement regarding this issue. Potentially, the recently published Doha agreement on the diagnosis and terminology in athletic groin pain [8] would help move clinical practice and research forward by enabling more robust results collation via shared nomenclature.

Measurement protocols for each specific movement and muscle function variable also varied across the included studies. For example, for the measurement of adductor muscle strength, three studies used hand-held dynamometers [18, 21, 28], two used sphygmomanometers [25, 27] and one used an isokinetic dynamometer [23]. Additionally, one study using a hand-held dynamometer used it in two contraction types: isometric and eccentric [18]. Further research is needed on the validity of each measure and a consensus on the optimal methods would again improve both research synthesis and clinical translation. Additionally, variation in outcome measures and methodology across included studies limited the potential for data pooling.

Although we included only studies investigating movement and muscle function factors in athletic populations, this included varied sports disciplines and participation levels. This is both a strength and a potential weakness of our synthesis, as data pooling in such heterogeneous groups entails combining results from cohorts who have different sports-specific training and participation volume. While these factors are highly likely to influence the injury risk and presentation profile, it was nonetheless judged that the pooling conducted was valuable to strengthen the review findings, considering the paucity of research currently available for each group. This may need to be re-considered once the volume of work is sufficient at different sporting levels and in different disciplines.

Interpreting the results of prospective studies was complicated by a lack of methodological clarity in manuscripts; for example, testing dominant or non-dominant limbs, moving or not moving, left or right, and injured or uninjured [13, 17, 22, 29, 33]. The most accessible approaches [30, 32] clearly measured and compared dominant and non-dominant sides. Additionally, only some retrospective studies were clear about the side of measurements [18, 19, 21, 26, 27, 31]. Given that unilateral symptoms can reflect bilateral biomechanical dysfunction, it would be our recommendation that future work examines movement on both sides, under any and all conditions assessed, and analyses data with reference to both symptom and dominance. In this review, however, we chose to analyse the data from the dominant or right leg only, to maintain the consistency of the analysis despite different ways of presenting the data by individual authors.

Very few retrospective studies attempted to blind the measurement assessor [18, 25, 29, 32] and only one study reported detailed sample size and power calculations [27].

Five studies [19, 22, 26, 29, 31] did not report the reliability of the measurements in the assessors’ hands. Addressing these methodological limitations in future research is needed to improve confidence in findings, and subsequently in the ‘levels of evidence’ that can be concluded.

Surprisingly, some studies [13, 20, 22, 29, 31, 33] did not provide basic anthropometric data such as age, height and weight, which limits the external applicability of findings and can be critical confounding factors, or co-variates, in biomechanical research. In particular, factors such as strength and muscle activation may clearly depend on the individual athlete’s fitness and muscle morphology. To avoid a potentially significant source of bias, all studies investigating biomechanical factors should accurately measure these factors and include them in the analysis. Additionally, differences in participants’ sex as well as pelvis width and tilt may be confounding factors as they significantly affect the loading [36, 37] and the biomechanics of the area, which may bias the individual study results.

### Coronal Plane Muscle Activation and Strength

#### Adductor Muscles

There is common agreement that the main muscles affected by SRGP are the hip adductors [7, 20], an assertion confirmed by 11 studies reporting decreased adduction strength associated with groin pain symptoms. Overall, there is strong evidence of an association between adductor muscle weakness and SRGP. Meta-analysis results showed strong evidence of adductor muscle weakness after SRGP onset, but only when measured by the squeeze test in 45° of hip flexion. This may indicate the importance of testing the groin symptoms using this particular test, which seems most sensitive to detect strength deficits in athletes with SRGP. There was limited evidence of decreased adduction strength prior to SRGP onset. It is important to note that there were four other prospective studies [20, 22, 29, 33] reporting adductor muscle weakness prior to the onset of SRGP, but presentation of the data in those studies did not allow for data pooling. Adductor muscle weakness in the pre-season was associated with SRGP onset indicating that strengthening of this muscle group may be a key component of prevention. Crow et al. [20] reported decreased adductor muscle strength 2 weeks prior to SRGP onset, but no earlier, suggesting a potential neuro-inhibitory mechanism for altered adductor motor output immediately before or at the time of pain onset for some athletes rather than long-standing weakness. Clinicians should consider implementing prevention strategies based on adductor strength screening findings.

Six studies investigated the association between abduction flexibility and SRGP [17, 18, 22, 25, 27, 33] and only one retrospective LQS reported a significant association [27]. However, pooled results show moderate evidence that abduction flexibility was not changed before, but increased after SRGP onset, measured with the bent knee fall-out test.

The reason for such changes is not clear. There may be a relationship between optimal hip abductor flexibility and SRGP, with too much flexibility being problematic. It is worth noting, however, that the flexibility increase was noted only during the bent knee fall-out test, which is a combination of abduction and external rotation flexibility test. It is possible that this flexibility increases following pain onset, removing the compensations for adductor weakness prior to symptom onset. Further, there may be an interaction between joint load, increased flexibility and sports participation volume. Further research is needed to elucidate the relationship between these factors, with such work having the potential to clarify aetiology.

#### Abductor Muscles

There is a commonly held belief that SRGP might be at least partly owing to muscle imbalance in the pelvic girdle area and, consequently, sub-optimal loading on groin structures [26, 38]. There is an association between decreased hip abduction strength and SRGP observed in prospective, but not retrospective studies [18, 23, 25, 30]. It is plausible that there is a weakness of hip abductors preceding SRGP onset that disappears following pain onset or subsequent rehabilitation. This rehabilitation may be particularly important for the GM muscle, which is thought to have a primary stabilising function [39], and should be considered in future research.

#### Relationship Between Abductor and Adductor Muscles

A prospective study by Tyler et al. [22] reports a significant decrease in adduction in relation to abduction strength associated with SRGP in professional (ice hockey) players, while Morrissey et al. [26] found a decrease in the GM-to-AL activation ratio in amateur footballers. The relationship between muscle strength and activation is not linear [40]. Therefore, although seemingly contradictory, if the abductor muscles are weaker they may need to increase activity to achieve their function of pelvic girdle stability. Additionally, GM activity was measured during a standing hip flexion movement (a functional task), whereas strength measurements were obtained using a maximal voluntary contraction break test and an isolated hip abduction task [22]. These measures clearly investigate different aspects of the strength construct in a functional vs. non-functional task. Research designs that include muscle activation in functionally relevant tasks and strength measures are needed to broaden our understanding of how different aspects of muscle function can be affected in SRGP.

### Horizontal Plane Hip Movement

Strong evidence of a decrease in hip total external rotation ROM after the SRGP onset was the only significant finding in horizontal plane hip movements. It is not clear whether this ROM limitation has muscular or articular origin, and there might be a number of reasons why it exists. For example, hip rotation restriction may follow increased hip joint loading owing to muscle imbalance around the hip (e.g. reduced abductor strength). Decreased ROM in athletes may also be related to underlying hip joint injury, which may be asymptomatic. Limitation of rotation ROM is clearly an area that requires further research in athletes with SRGP, as a clear distinction needs to be made between articular and muscular movement restrictions.

### Other Muscle Function and Architectural Features

A decrease in TrA thickness and delayed onset during movement was found to be associated with SRGP. Cowan et al.’s HQS reported delayed TrA activation in relation to the ‘prime mover’ in a straight leg raise manoeuvre [19], while Jansen et al. reported a reduced relaxed cross-sectional area [21]. These findings suggest that muscle dysfunction in SRGP is not limited to hip muscles and TrA function may be an important prevention and rehabilitation consideration in some affected athletes. While two HQS may not be enough to draw a strong association with SRGP, it is important to remember that abdominal-related groin pain has been long established as a major source of SRGP [7, 8]. In this context, the paucity of research focussing on the abdominal muscles is even more surprising, and suggests a broad area for further research.

### Clinical Implications and Future Directions

In this section, we summarise the muscular and movement alterations associated with SRGP that could be considered during the development of rehabilitation and prevention programmes. The strongest prospective risk factor from this review was reduced hip adductor strength, which should be considered for inclusion in pre-season screening programmes. There is some indication for more regular screening of adductor strength in some environments (e.g. elite sport) given it may precede pain onset by 2 weeks in some individuals who then develop SRGP [20], although further studies in elite and other athletic populations are needed to confirm this finding. Recommendations for adductor muscle strength measurement and treatment strategies are well described. They include squeeze and unilateral resisted adduction tests to establish any potential strength deficits, which are suggested to be clinically relevant with an over 10 % strength difference between two limbs [41, 42]. In all of the reviewed studies, the difference between the injured and uninjured players was over 10 %, ranging from 14 to 28.5 %. Additionally, various exercises of graduated difficulty are proposed to restore them, such as squeezing the ball between knees in the early phase of rehabilitation and moving to long lever (ball between the feet) and open kinetic chain strengthening exercises using resistance devices as rehabilitation progresses [43, 44]. Other factors preceded groin pain onset but the evidence was limited. These included decreased hip abductor muscle strength, and decreased knee extensor strength, indicating screening for and addressing identified deficits may reduce the incidence of SRGP. The most effective interventions for addressing hip and knee muscle function deficits and whether they decrease the incidence of groin pain warrant further investigation. Restriction in hip external rotation ROM, in athletes with SRGP, may be critical owing to the requirement for a sufficient range of hip movement for adequate load absorption during change of direction activities [45]. Clinicians should identify whether the underlying cause of possible deficits in hip rotation ROM is articular or muscular. If muscular restriction is present, specific techniques including stretching, soft-tissue work as well as using the entire ROM in sports-specific tasks during the end phase of rehabilitation should be considered. Articular restriction may be less likely to change with these interventions, and end range loading may even provoke symptoms [46]. This may partly explain why addressing flexibility specifically (e.g. stretching, soft-tissue techniques) is less of a feature of current groin rehabilitation and prevention programmes than adductor and other muscle strengthening [4, 5, 43].

This review has highlighted that there are very few studies that have investigated muscle activation and timing deficits during functional movement tests in subjects with SRGP. Gross maximal voluntary contraction tests may not be sensitive enough to identify subtle motor output deficits. The assessment and treatment options for potential pelvic movement control deficits are not well established and certainly require further investigations. The authors of this review recommend careful clinical assessment of functional movements such as standing hip flexion [26] or single leg squat, which reflect common frequent movements in sports possessing a high incidence of SRGP. Additionally, sport-specific movements (e.g. cutting) should also be evaluated; with a particular focus on the reliability and clinical applicability of the functional testing.

There is therefore clearly a need to investigate pelvic girdle muscle characteristics during functional tasks, in various groups of athletes. For example, no study has prospectively investigated abdominal muscle characteristics as a risk factor for SRGP, which should be prioritised as a research goal given the clear association with existing symptoms. Similarly, prospective studies should address hip adductor, hip abductor and knee extensor muscle strength; as well as hip rotation ROM change prior to SRGP.

## Conclusions

Our review identified a ROM and muscle function features that can be prospectively identified in a range of athletes who subsequently develop SRGP and should be considered in screening programmes (Tables 3, 4). These findings provide clear clinical guidance that should be implemented in the prevention and rehabilitation of athletes with SRGP.

**Table 4:** Summary of features, findings and levels of evidence for all studies included in the review

Muscle	Feature	Prospective/retrospective	Studies not included in pooled results	Studies included in pooled results	Included studies quality	Specific criterion	Pooled result/calculated SMD	Evidence
Adductor	Strength	Prospective	Emery and Meeuwisse [33]	O’Connor [30]	HQS	Isokinetic hip adduction in 0.52 rad*s^−1^	No change	Limited evidence
			Engebretsen et al. [29]	O’Connor [30]	HQS	Isokinetic hip adduction in 2.08 rad*s^−1^	Decrease in SRGP	Limited evidence
			Crow et al. [20]					
			Tyler et al. [22]	O’Connor [30]	HQS	Isokinetic hip adduction in 3.66 rad*s^−1^	No change	Limited evidence
		Retrospective		Malliaras et al. [25]	HQS	Squeeze test 0°	Decrease in SRGP	Limited evidence
				Malliaras et al. [25]	HQS	Squeeze test 30°	Decrease in SRGP	Limited evidence
				Jansen et al. [21]	HQS	Squeeze test 45°	Decrease in SRGP	Strong evidence
				Malliaras et al. [25]	HQS			
				Mens et al. [28]	HQS			
				Nevin and Delahunt [27]	LQS			
				Mohammad et al. [23]	LQS	Isokinetic concentric hip adduction	No change	Very limited evidence
				Thorborg et al. [18]	HQS	Isometric hip adduction	No change	Limited evidence
				Thorborg et al. [18]	HQS	Eccentric hip adduction	Decrease in SRGP	Limited evidence
	Flexibility	Prospective	Emery and Meeuwisse [33]	Arnason et al. [17]	HQS	Unilateral abduction flexibility test	No change	Moderate evidence, not homogeneous
				Tyler et al. [22]	HQS			
		Retrospective		Malliaras et al. [25]	HQS	Right bent knee fall out	Increase in SRGP	Moderate evidence
				Nevin and Delahunt [27]	LQS			
				Thorborg et al. [18]	HQS	Unilateral abduction flexibility test	No change	Limited evidence
	Peak torque angle	Prospective		O’Connor [30]	HQS	Peak torque angle	No change	Limited evidence
Abductors	Strength	Prospective		O’Connor [30]	HQS	Isokinetic hip abduction in 0.52 rad*s^−1^	Decrease in SRGP	Limited evidence
				O’Connor [30]	HQS	Isokinetic hip abduction in 2.08 rad*s^−1^	No change	Limited evidence
				O’Connor [30]	HQS	Isokinetic hip abduction in 3.66 rad*s^−1^	No change	Limited evidence
		Retrospective		Malliaras et al. [25]	HQS	Isometric hip abduction	No change	Strong evidence
				Thorborg et al. [18]	HQS			
				Mohammad et al. [23]	LQS	Isokinetic concentric hip abduction	No change	Very limited evidence
Relationship between abductor and adductor muscles	Strength	Retrospective		Mohammad et al. [23]	LQS	Isokinetic concentric hip adductor vs. abductor strength	No change	Very limited evidence
				Tyler et al. [22]	HQS	Isometric hip adductor vs. abductor strength	Decrease in SRGP	Limited evidence
	Activation	Retrospective		Morrissey et al. [26]	HQS	Moving leg: early phase of SHF	Decrease in SRGP	Limited evidence
				Morrissey et al. [26]	HQS	Moving leg: middle phase of SHF	No change	Limited evidence
				Morrissey et al. [26]	HQS	Moving leg: late phase of SHF	No change	Limited evidence
				Morrissey et al. [26]	HQS	Weight-bearing leg: early phase of SHF	Decrease in SRGP	Limited evidence
				Morrissey et al. [26]	HQS	Weight-bearing leg: middle phase of SHF	Decrease in SRGP	Limited evidence
				Morrissey et al. [26]	HQS	Weight-bearing leg: late phase of SHF	Decrease in SRGP	Limited evidence
Hip flexors	Strength	Retrospective		Mohammad et al. [23]	LQS	Isokinetic concentric hip flexion	Increase in SRGP	Very limited evidence
				Thorborg et al. [18]	HQS	Isometric hip flexion	No change	Limited evidence
				Thorborg et al. [18]	HQS	Eccentric hip flexion	No change	Limited evidence
	Flexibility	Prospective		Arnason et al. [17]	HQS	Modified Thomas’s test	No change	Limited evidence
		Retrospective		Thorborg et al. [18]	HQS	Modified Thomas’s test	No change	Limited evidence
Hip extensors	Strength	Retrospective		Mohammad et al. [23]	LQS	Isokinetic concentric hip extension	No change	Very limited evidence
Relationship between flexor and extensor muscles	Strength	Retrospective		Mohammad et al. [23]	LQS	Isokinetic concentric hip flexion vs. extension	No change	Very limited evidence
Hip rotation ROM	Hip internal rotation	Prospective	Ibrahim et al. [13]	Verral et al. [32]	LQS	Passive hip internal rotation test	No change	Very limited evidence
		Retrospective		Nevin and Delahunt [27]	LQS	Passive hip internal rotation test	No change	Strong evidence
				Thorborg et al. [18]	HQS			
				Malliaras et al. [25]	HQS			
				Malliaras et al. [25]	HQS	Passive total hip internal rotation (sum of both legs)	No change	Moderate evidence
				Verral et al. [31]	HQS			
	Hip external rotation	Prospective	Ibrahim et al. [13]	Verral et al. [32]	LQS	Passive hip external rotation test	No change	Very limited evidence
		Retrospective		Nevin and Delahunt [27]	LQS	Passive hip external rotation test	No change	Strong evidence
				Malliaras et al. [25]	HQS			
				Malliaras et al. [25]	HQS	Passive total hip external rotation test (sum of both legs)	Decrease in SRGP	Strong evidence
				Verral et al. [31]	HQS			
Knee extensor	Strength	Prospective		O’Connor [30]	HQS	Isokinetic knee extension	No change	Limited evidence
Knee flexor	Strength	Prospective		O’Connor [30]	HQS	Isokinetic knee flexion	Decrease in SRGP	Limited evidence
Transversus abdominis	Thickness	Retrospective		Jansen et al. [21]	HQS	Resting thickness: right-sided symptoms	Decrease in SRGP	Limited evidence
						Resting thickness: left-sided symptoms	Decrease in SRGP	Limited evidence

*ROM* range of movement, *HQS* high-quality study, *LQS* low-quality study, *SMD* standardized mean difference, *SHF* standing hip flexion, *SRGP* sports-related groin pain

Hip adductors and knee flexor strength deficits should be mainly screened and addressed as they may be risk factors for SRGP.

Further, this review identified both muscle function features and ROM considerations, clearly shown by retrospective studies that should be considered in rehabilitation programmes (Tables 3, 4). In particular, adductor muscle weakness and increased abduction flexibility, hip total external rotation deficits, imbalances between adductor and abductor muscles, increased hip flexor strength and transversus abdominis muscle thickness should be addressed in rehabilitation programmes. The lack of consistency about various classification issues, alongside methodological heterogeneity also need to be addressed to optimally move the evidence base forward.
